# Zero retinal vein pulsation amplitude extrapolated model in non-invasive intracranial pressure estimation

**DOI:** 10.1038/s41598-022-09151-7

**Published:** 2022-03-25

**Authors:** W. H. Morgan, A. Vukmirovic, A. Abdul-Rahman, Y. J. Khoo, A. G. Kermode, C. R. Lind, J. Dunuwille, D. Y. Yu

**Affiliations:** 1 Lions Eye Institute, Centre for Ophthalmology and Visual Science, University of Western Australia, Perth, Australia; 2 Centre for Neuromuscular and Neurological Disorders, Perron Institute AU, University of Western Australia, Perth, WA Australia; 3 Institute for Immunology and Infectious Disease, Murdoch University, Perth, WA Australia; 4 Neurosurgical Service of Western Australia, Sir Charles Gairdner Hospital, Nedlands, WA Australia; 5 Medical School, University of Western Australia, Perth, Australia; 6 Department of Ophthalmology, Counties Manukau DHB, Auckland, New Zealand; 7 Department of Neurology, Sir Charles Gairdner Hospital, Nedlands, Australia

**Keywords:** Medical research, Neurology

## Abstract

Intracranial pressure (ICP) includes the brain, optic nerve, and spinal cord pressures; it influences blood flow to those structures. Pathological elevation in ICP results in structural damage through various mechanisms, which adversely affects outcomes in traumatic brain injury and stroke. Currently, invasive procedures which tap directly into the cerebrospinal fluid are required to measure this pressure. Recent fluidic engineering modelling analogous to the ocular vascular flow suggests that retinal venous pulse amplitudes are predictably influenced by downstream pressures, suggesting that ICP could be estimated by analysing this pulse signal. We used this modelling theory and our photoplethysmographic (PPG) retinal venous pulse amplitude measurement system to measure amplitudes in 30 subjects undergoing invasive ICP measurements by lumbar puncture (LP) or external ventricular drain (EVD). We estimated ICP from these amplitudes using this modelling and found it to be accurate with a mean absolute error of 3.0 mmHg and a slope of 1.00 (r = 0.91). Ninety-four percent of differences between the PPG and invasive method were between − 5.5 and + 4.0 mmHg, which compares favourably to comparisons between LP and EVD. This type of modelling may be useful for understanding retinal vessel pulsatile fluid dynamics and may provide a method for non-invasive ICP measurement.

## Introduction

Intracranial pressure (ICP) is the pressure in the intracranial cavity determined by the measurement of cerebrospinal fluid (CSF) pressure. It is currently measured by tapping the cerebrospinal fluid space which is known to be generally equivalent to brain tissue pressure^1^ and so the terms CSF pressure and ICP are used interchangeably in most publications and throughout this paper. It is a fundamental physiological parameter in neurological disease but is poorly understood, partly because of its measurement difficulty^2^. Intracranial pressure is elevated in several common neurological disorders: traumatic brain injury, intracranial haemorrhage, brain tumour and idiopathic intracranial pressure. Normal ICP, measured by lumbar puncture in the lateral decubitus position, is considered between 7.4 to 14.7 mmHg (10–20 cmH_2_O), with pressures above 18.4 mmHg (25 cmH_2_O) being considered pathological^3^. The range between 14.7 and 18.4 mmHg is considered borderline.

Current techniques for measuring intracranial pressure utilize either a burr hole through the skull with external ventricular drain (EVD), pressure transducer implantation, or cannulation of the lumbar cerebrospinal fluid space via lumbar puncture (LP). The burr hole implantation techniques allow a continuous and dynamic pressure measurement over time. These can be calibrated and zeroed in-situ and are considered the most accurate. They are commonly performed in anaesthetised, critically ill patients with abnormal CSF pressure physiology. They are, unfortunately, prone to infection, haemorrhage and other complications^4^. It is difficult to repeat these particular measurements frequently due to their invasive nature. They are ideal for identifying ICP variations over different physiological conditions such as with posture and diurnal variation. Lumbar puncture is generally performed in the lateral decubitus posture in the lower spine at the level of L4, L5 interspace. A small gauge needle is inserted and then connected to tubing with gradations to allow pressure measurement. The LP needle cannot be left in-situ for any significant period of time, so variation in ICP is difficult to measure with this technique. It is also prone to post-procedural headache, infection and haemorrhage^3^.

Gravity affects ICP so that when a subject lies down in the lateral decubitus posture the intracranial pressure at eye and lateral ventricle level rises by an average 10 mmHg^5^. Hence considering posture induced pressure change is important when comparing ICP derived from these two techniques. Lumbar puncture measurements assume a direct and unrestricted fluidic connection between the lumbar spinal space and the intracranial cavity. Measurements comparing lumbar puncture measurements to intracranial pressure measurements by EVD show high accuracy with standard deviation of measured differences being just 2.1 mmHg and 95% of differences falling between − 5.1 to + 2.6 mmHg^6^.

Several non-invasive forms of intracranial pressure assessment have been proposed^7,8^. They include tympanic membrane displacement^9^, and optic nerve sheath diameter^10^. Central retinal artery velocity changes with orbital pressure alteration shows promise with a standard deviation of the difference between LP measurement and this technique being 2.2–6.2 mmHg with 95% confidence intervals ± 4 to 12 mmHg^11,12^. Additionally, the sensitivity of detecting an ICP greater than 14.7 mmHg with this technique is 68% (specificity 84%)^13^.

Retinal vein pulsation is known to be influenced by ICP^14,15^. More recently it has been shown that the pulsatile ICP acts as a pulse wave generator, propagating pulse waves along the central retinal vein into the eye^16^. Additionally, the central retinal vein experiences a large pressure change as it exits the eye. When pressures in the intraocular compartment exceed that in the cerebrospinal fluid pressure compartment, a flow discontinuity environment is produced^17^. Recent engineering modelling of analogous situations reveals that retinal vein pulse amplitude equivalents rise in an almost linear manner as intraocular pressure (IOP) is elevated above the balance point between IOP and CSF pressure equivalents^18^. We have developed a photoplethysmographic (PPG) technique which can detect small shifts in green light transmission through vessels, in time with the cardiac cycle using a form of signal averaging and Fourier analysis^16^. Pulsation may not be visible in all subjects but the PPG signal can be detected with greater sensitivity than direct observation^19^, and pulsation becomes more visible as IOP is elevated^20^. Preliminary analyses suggesting that one can use pulse amplitude changes with IOP alteration to detect this balance point and hence ICP. We were interested in refining the vein pulse amplitude data selection and balance point detection to allow exploration of this relationship with ICP.

## Results

**Figure 1:**
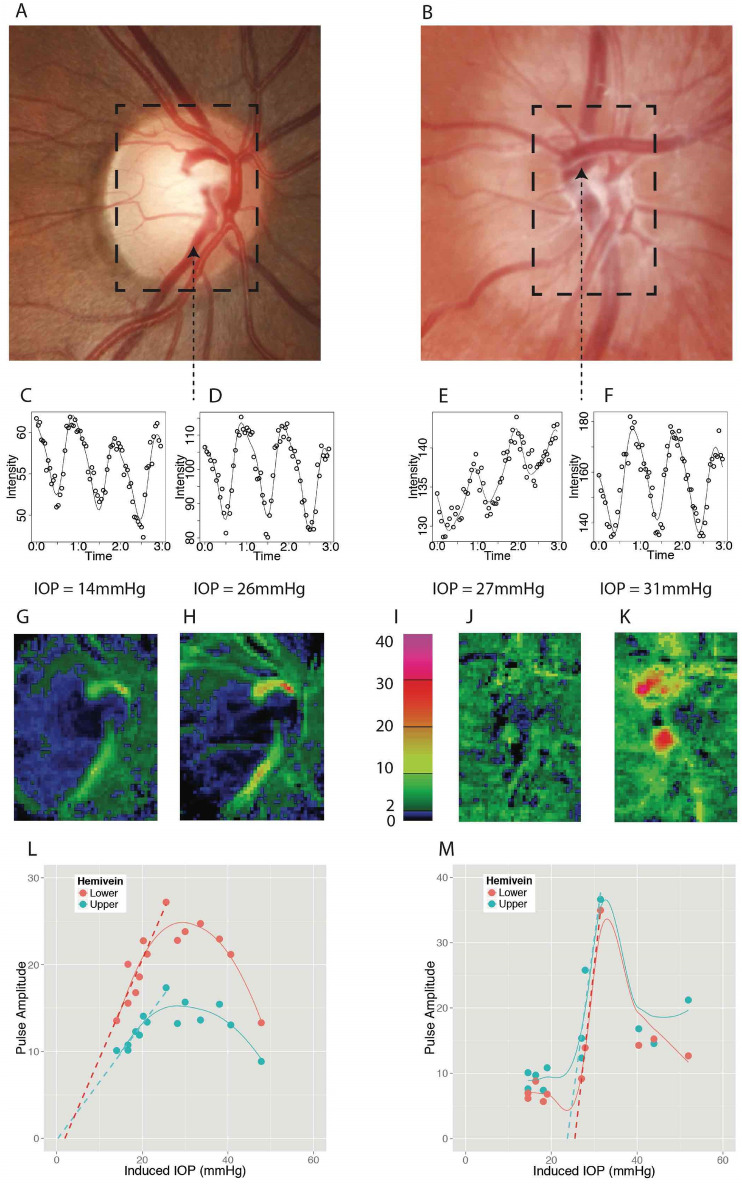
Schematic diagram explaining the analytical process of estimating ICP. Optic disk photographs of normal (7 mmHg, **A**) and elevated ICP (31 mmHg, **B**) eyes. The region of analytical interest (ROI) is shown in the dashed rectangles. Papilloedema can be seen in (**B**). Two harmonic curve fits are applied to data from all locations across the entire ROI with examples from one location at specified IOP shown in the inferior hemivein in (**C**) and (**D**) and superior hemivein in (**E**) and (**F**) (arrows). These locations were the sites of maximal pulse amplitude for the respective vessels. The pulse amplitudes in arbitrary units are calculated and mapped to the specified IOP to correspond with the ROI (**G**,**H**,**J**,**K**) using a false colour scale (**I**). The maximum pulse amplitude values from the upper and lower hemiveins are chosen automatically from the retinal hemiveins using the map and compared against induced IOP (**L**,**M**). Linear regression is applied to the rising part of the curve and extrapolated back through the x-axis coinciding with zero pulsation (dashed lines). This value becomes the estimated ICP in mmHg. In (**L**) it is 1 mmHg in erect posture equivalent to 11 mmHg in lateral decubitus. In (**M**) it is 24 mmHg erect posture equivalent to 34 mmHg lateral decubitus. The general shape of the curve can be seen from the locally weighted fit (solid lines).

**Figure 2:**
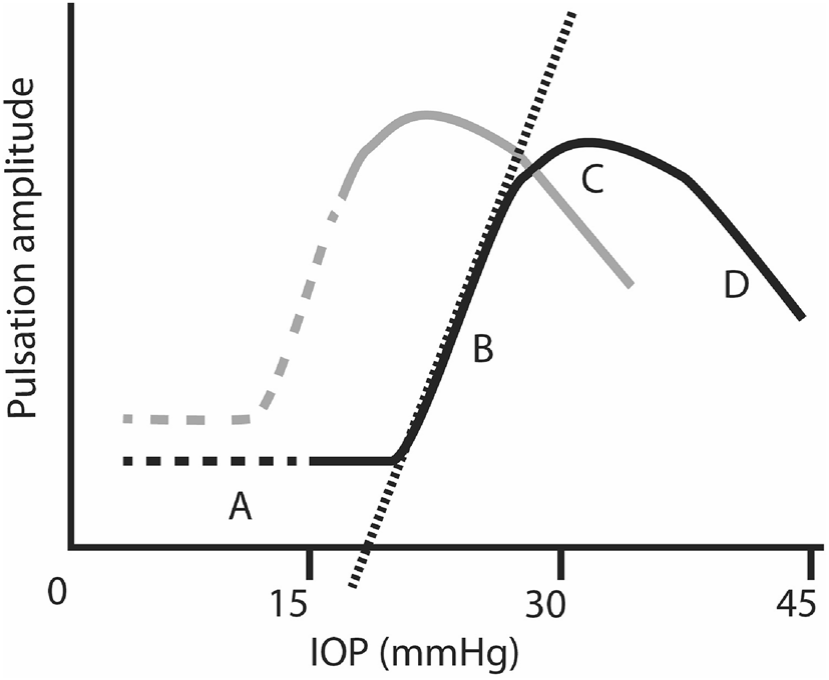
Schematic diagram of typical maximum pulse amplitude vs intraocular pressure curve. It is not usually possible to gain data from IOP below 15 mmHg so that is presumed and shown as dashed line. The black curve represents data from a subject with an elevated ICP of 19 mmHg, having a flat initial phase (**A**) then rising (**B**) to a peak (**C**) which then starts to drop (**D**) as IOP increases. Analysis aims to extract data from the rising segment (**B**), perform linear regression and extrapolate (dotted line) it back to the x-axis, where amplitude tends to zero and theoretically ICP = IOP. When ICP is lower (grey line), the curve shifts to the left and there may be limited data available in the rising phase for regression analysis. So data with a maximum slope less than 0.4 au/mmHg is excluded.

Thirty-five subjects who had direct ICP measurement within 3 days were examined, one of who was examined twice, 7 months apart. Two subject recordings were excluded due to poor video recordings and 3 were excluded due to flat amplitude slopes without a significant downslope greater than 0.4 arbitrary units (au)/mmHg or maximum amplitudes were less than 6 au. All of the latter three subjects had clinical papilloedema with ICP measured by lumbar puncture of 22.1, 18.4 and 5.9 mmHg. The 2 former subjects were diagnosed with IIH and the latter with papillitis. The remaining 30 subjects (28 female) had a mean age of 34 (sd 13) years. Fourteen have been reported on before using our prior, less selective analytical algorithm^21^. Twenty eight were referred from neurology being investigated for possible idiopathic intracranial hypertension. Two were from the neurosurgery high dependency unit with external ventricular drains in situ following subarachnoid haemorrhage. The rising phase in amplitude vs IOP was detected by the 3 phase algorithm then calculating the estimated ICP (Fig. 1). The general form of the relationship is shown in Fig. 2.

**Figure 3:**
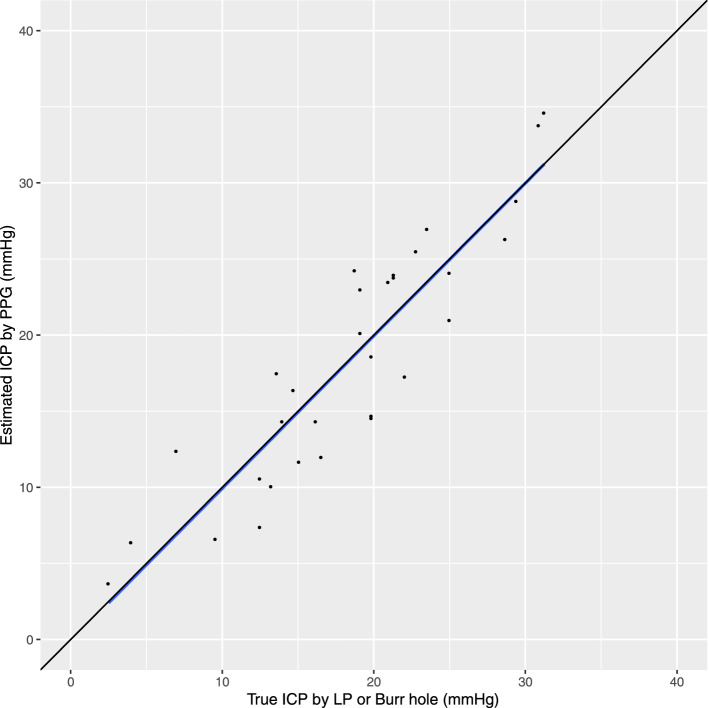
Association between ICP in mmHg measured by either external ventricular drain (EVD) or lumbar puncture (LP) and the estimated ICP calculated by photo-plethysmography (PPG). The line of best fit is shown in blue with the 95% confidence interval (shaded area). The line of perfect unity is also shown (black).

The mean direct ICP was 18.4 mmHg (sd 7.2) ranging from 2.5 to 30.9 mmHg. The lowest 2 measures (2.5 and 4.0 mmHg) were both from patients with EVD in the sitting posture. All others were by lumbar puncture in the lateral decubitus posture. The direct measurement by LP occurred a mean 1.6 (sd 1.0) days after PPG measurement. The relationship between estimated and direct measurement of ICP was strong with a correlation coefficient of 0.91 (Fig. 3). The slope was 1.00 (p < 0.0001) with a standard error of 0.09 and a 95% confidence interval of 0.82–1.18.

**Figure 4:**
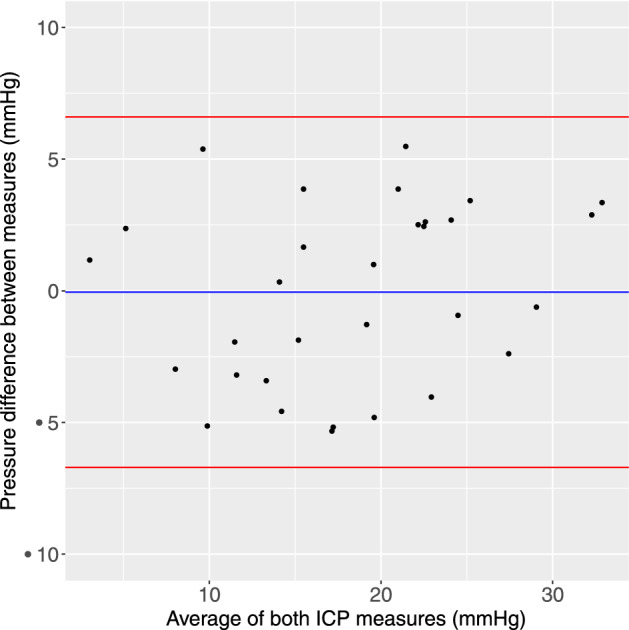
Bland–Altman graph shows the difference between estimated and ‘true’ ICP vs the average of the two pressure estimates. The mean is shown (blue line) as well as ± 1.96 standard deviations (red lines).

**Figure 5:**
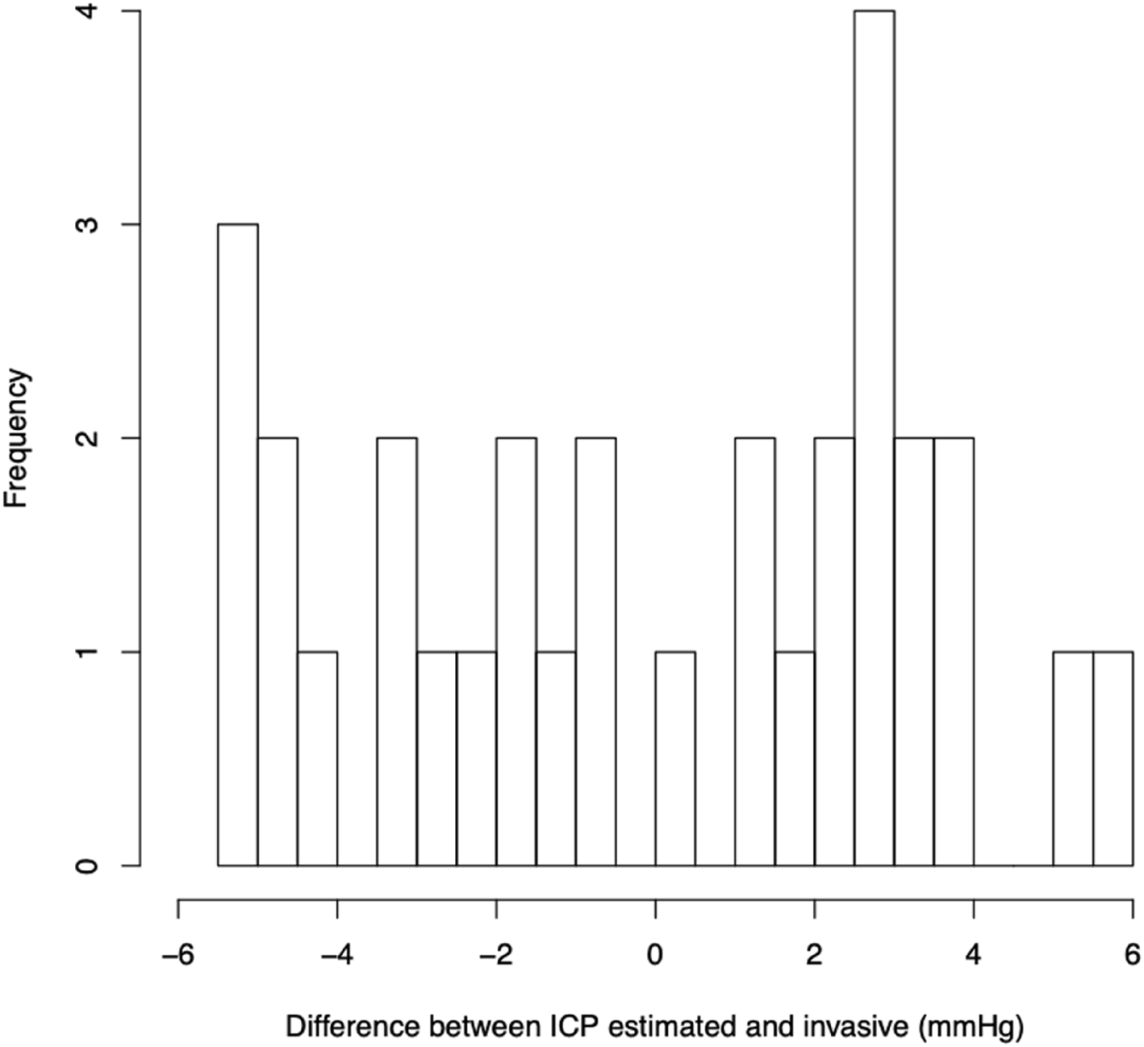
Frequency histogram showing the distribution of the differences between estimated and invasive ICP measurements.

Estimates of accuracy were calculated. The mean difference between invasive (LP and EVD) and PPG measures of ICP was − 0.05 mmHg (sd 3.2, Fig. 4) with 95% confidence interval of the mean was between − 2.2 to + 2.1 mmHg. The Bland–Altman 95% confidence intervals for differences was − 6.3 to 5.8 mmHg. The mean absolute difference between invasive and PPG measures was 3.0 mmHg (sd 1.5). Ninety-four percent of differences were between − 5.5 and + 4.0 mmHg (Fig. 5). The mean absolute difference between measures in the 2 EVD patients was 0.95 mmHg. The distribution of the differences between direct and estimated measures was platykurtic with a kurtosis of 1.69 (Fig. 5), little skewness (− 0.10) and was acceptably normally distributed (Shapiro–Wilk 0.93, p = 0.053).

The sensitivity of PPG for detecting a borderline elevated ICP measured invasively greater than 14.7 mmHg (20 cm H_2_O) was 91% with specificity of 89%. The sensitivity for detecting an elevated ICP measured invasively greater than 18.4 mmHg (25 cmH_2_O) was 83% with specificity of 100%. In the latter group, all 3 false negatives had a non-invasive ICP measurement greater than 14.5 mmHg, with the other subject having a PPG measurement of 14.6 mmHg.

## Discussion

This PPG method demonstrates that the vein pulse amplitude relationship to intraocular pressure is similar to that modelled assuming flow along a collapsible channel from a high pressure chamber to a lower pressure^18,22^. This theoretical model predicts that where amplitude tended to zero along the rising phase of the curve, the chamber pressure (IOP) should equal downstream pressure in the optic nerve subarachnoid space, which may generally be equivalent to ICP. That this value had a strong correlation with invasive ICP measures supports the use of flow discontinuity modelling for understanding venous flow dynamics in this region. This technique may allow noninvasive ICP measurement across the physiological and elevated ICP range. The mean difference between estimated and direct ICP of − 0.05 mmHg (sd 3.6) was comparable to that between lumbar puncture and EVD (− 0.75, sd 2.1 mmHg)^6^. The range and distribution of 94% of observed PPG differences being between − 5.5 to + 4 mmHg was similar to that of 95% of EVD and lumbar puncture differences being between − 5.1 to + 2.6 mmHg^6^. The Bland–Altman 95% confidence intervals of − 2.3 to + 1.8 mmHg are similar to or lower than those reported by other non-invasive ICP techniques using central retinal artery ultrasound (± 4 to 12 mmHg)^11,12^. This PPG method has a high sensitivity and specificity for detecting elevated ICP and could be clinically useful, particularly in this particular cohort consisting mainly of IIH patients.

The altered algorithm from that initially described^21^, has resulted in some improvement in specificity for detecting elevated ICP (89% vs 80%) as well as reduction in mean differences to − 0.05 mmHg, from − 0.35 mmHg. The regression slope between invasive and non-invasive measures was 1.00 compared to 0.92, which could simply reflect the increase in data but does support the use of this collapsible vessel model for predicting ICP.

Most IIH patients are ambulatory and able to concentrate for a significant period during examination. This photoplethysmography technique appears well suited for this particular diagnostic group. It may be useful for follow-up and helping to inform clinical progress and response to therapy. It may also help avoid multiple lumbar punctures with their morbidities. Patients with intracranial mass lesions and Chiari malformation are at greater risk of brainstem herniation after lumbar puncture^23,24^. This PPG technique may be useful in these and other situations where there is a higher risk of morbidity with lumbar puncture.

A significant limitation of this study is the fact that the lumbar puncture was not performed at exactly the same time as photoplethysmography so the time delay and possible changes in ICP between video recording and lumbar puncture may add to the differences between pressure estimates. Several sources of error may affect the accuracy of this technique including measuring and converting the ophthalmodynamometric force to IOP as well as the use of a fixed 10 mmHg pressure difference conversion from lateral decubitus to sitting posture. The difference is likely to vary according to subject torso length inducing variable gravitational affects upon postural CSF pressure differences. The several sources of error may combine to partially explain the platykurtic distribution of pressure differences.

This technique requires that a measurable change in pulsation amplitude occurs above baseline amplitude. Three (8%) of measurements were excluded due to this, all of whom had advanced papilloedema which we assume was compressing the central retinal vein. The analysis can detect this and along with clinical signs alert the observer to the fact that elevated intracranial pressure is likely. Two (6%) had poor quality recordings preventing the analysis. This 6% figure may be reflective of the proportion of the ambulatory, awake population in whom this technique may not be suitable. The data suggests that in 86% of the population relatively accurate estimates of ICP can be gained noninvasively with this technique and that in an additional 8% the analysis will alert the clinician to the likely presence of papilloedema. The main cause of low pulse amplitudes along a range of IOP is a narrowed central retinal vein^16^. Other causes of central retinal vein narrowing include central retinal vein occlusion and severe glaucoma, which should be recognisable clinically at the time of examination^25^. We have only used maximal pulse amplitude data and have not used vessel location or timing information. These data, along with pulse amplitude distribution data, can all be derived from this photoplethysmography analysis and may help improve intracranial pressure assessment measurement accuracy.

## Methods

The use of human subjects for the PPG measurements was approved by Belberry Human Research Ethics Committee under permit number 2015-11-756-A-2, in accordance with the declaration of Helsinki and in compliance with National Health and Medical Research Council guidelines for clinical trials. All measurements were performed according to relevant guidelines and regulations and informed consent was obtained from all participants. All measurements were obtained using standard clinical protocols for equipment cleaning and best practice following Lions Eye Institute COVID guidelines.

Neurosurgical and neurology patients were recruited who were undergoing intracranial pressure assessment and who could perform photo-plethysmography (PPG). Neurosurgical patients were those in the neurosurgery high dependency unit who had implanted EVD monitors with continuous measurement of intracranial pressure. They were required to be well enough to be able to sit at the side of their bed and undergo slit lamp examination with contact lens ophthalmodynamometry. Neurology patients were those being investigated for possible idiopathic intracranial hypertension undergoing lumbar puncture electively. All neurology patients were seen and examined between zero and three days prior to their lumbar puncture in order to minimize the time delay between PPG measurement and lumbar puncture. All subjects were assessed by members of their clinical unit for fitness to undergo the required measurements before being invited to participate in this study.

**Figure 6:**
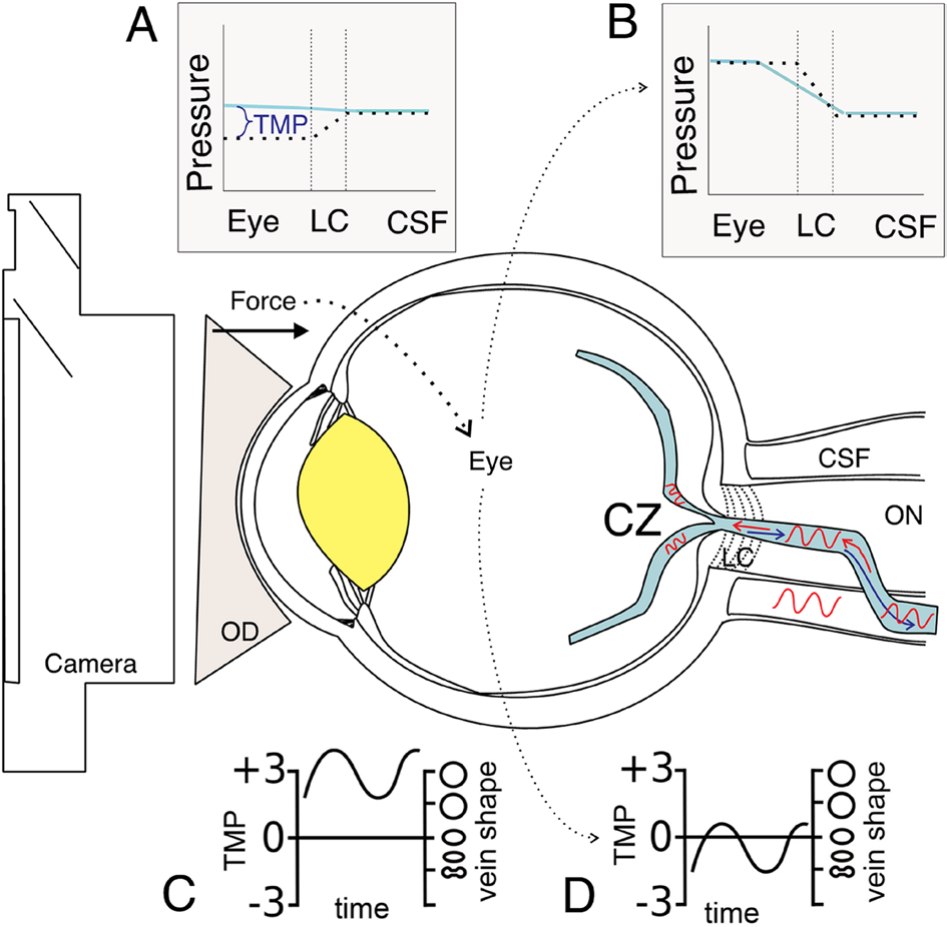
Schematic diagram of experimental setup. Cerebrospinal fluid pressure (CSF) pulse generates central retinal vein pulsation, which propagates retrograde (red) to flow (blue) towards the central zone (CZ) on the optic disk surface, then into upper and lower hemiveins on the disk and retinal surface. When CSF pressure is elevated above eye pressure (IOP—eye) the venous pressure (blue line, **A**) will be greater than surrounding tissue pressure (dashed line **A**) in the eye and lamina cribrosa (LC). This leads to a high transmural pressure (TMP in **C**) which results in minimal vein shape change over the pulse pressure range due to the venous compliance characteristics (**C**). If an ophthalmodynamometer is applied (OD) with some force, eye pressure can be elevated to balance or exceed CSF pressure. In (**B**) it exceeds CSF pressure with a pressure drop across the LC resulting in a lower TMP favouring significant and visible venous shape change (pulsation) across the pulse pressure range (**D**). A video camera can record the venous pulsation.

The photoplethysmography technique undertaken in this paper is described in more detail^16,19^. In brief, it allows the pressure gradient across the lamina cribrosa to be altered while recording vessel pulsation, which is amplified when the IOP starts to exceed ICP because of the non-linear venous wall response to transmural pressure (Fig. 6). All patients underwent a full ophthalmic examination including intraocular pressure (IOP) measurement and dilation with retinal examination to exclude other non-ICP related pathologies such as central retinal vein occlusion and glaucoma. Intraocular pressure measurement was performed using Goldmann applanation tonometry. Ophthalmodynamometry was performed using a Meditron ophthalmodynamometer (Meditron, Volklingen, Germany, Fig. 6), which in principle consists of a three-mirror Goldmann lens attached to a ring force transducer allowing continuous force output. The calibration to calculate induced IOP was baseline IOP + 0.89 × force output^26^.

For the neurosurgical patients, the slit lamp camera was wheeled to the patient bedside and the patient examined at the bedside. The neurology patients examined were ambulatory and could attend the eye clinic for video-photography. For video-photography, all subjects had a pulse oximeter (Nellcor N65, Covidien, Mansfield, MA) placed on their right index finger with the audible oximeter ‘beep’ recorded during photography. They all sat at the slit lamp camera (Carl Zeiss, Germany) while video recordings were taken at 25 fps (Canon 5D mark III, Canon Corp, Japan). At least 3 consecutive cardiac cycle length recordings were taken at each IOP setting with the pressure being applied in approximately 5 mmHg steps from baseline to 45 mmHg.

The green colour channel video frames are aligned and each location set of intensity values are analysed over time using harmonic analysis (Fig. 1C–F) to calculate the blood vessel pulsation amplitude at each point on the retina. The amplitude values are measured in arbitrary units using a derivation from the Beer–Lambert law attempting to approximate the amplitude in microns^27^. A 2 dimensional matrix of these amplitude values corresponds to the anatomical distribution of vessels and can be displayed as a heat map (Fig. 1G–K). The retinal veins are identified manually on a corresponding image allowing retinal vein amplitude values to be extracted. The subsequent analysis was similar to that previously described with several significant differences^21^. The quality of the harmonic curve fit was assessed by using the probability of the first harmonic sin coefficient being greater than zero. We used only curve fit results where that probability was less than 0.03. The superior and inferior retinal veins were outlined and the maximum pulse amplitudes from each superior and inferior hemi-retinal vein were chosen for analysis. Any obscuration of the vessel limits the number of amplitude measures possible from that vessel, so the algorithm eliminated data sets that had less than twenty retained amplitude measures in each vessel. The signal to noise ratio reduces with eccentricity due to pulse wave attenuation^28^. So, the vessel analysis extent was limited to measures within the central 0.8 mm of disk centre to use amplitude measurements with higher signal to noise ratio.

These maximum pulse amplitudes were compared with induced IOP (Fig. 1L,M). Our initial work used a simple broken stick analysis^21^. With the analysis used in this study, we identified 3 phases of the curve: initial flat, rising and plateau to falling. This model was implemented through an algorithm which first identifies the value of IOP corresponding to peak maximal pulsation amplitude. Values corresponding to intraocular pressures greater than this level including the plateau to falling phase are eliminated. The remaining amplitude: IOP data pairs were grouped into all possible combinations of consecutive pairs with minimum 3 consecutive data pairs. Linear regression was then performed upon these combinations excluding any combinations with Pearson’s correlation coefficient less than 0.8 or slopes less than 0.4 units amplitude/mmHg. These combinations of datasets were required to have a maximum amplitude of at least 6 units. The line of best fit by least mean squares applied to the remaining dataset with highest slope was extrapolated back through the rising phase to the x-axis and this x-intercept value recorded. All this analysis was automated and completed in R^29^.

This x intercept was reported in mmHg and for subjects undergoing lumbar puncture, converted to a lateral decubitus (LD) position posture equivalent using the formula ICP_LD_ = ICP_sitting_ + 10 mmHg. This is based on the average difference in pressure between the two postures from the work by Magnaes^5^. Most patients had both upper and lower hemivein data from both eyes allowing 4 datasets to be available for analysis. A mean of these estimates was taken to be the estimated ICP.

### Statistical analysis

The estimated ICP was compared to direct measurement of ICP (LP or EVD) using linear regression, then by calculating the difference in these measures. The mean difference and standard deviation can be compared to other techniques. The mean of absolute differences was calculated. Bland Altman analysis was performed to give a graphical estimate of accuracy and also the 95% percent limits of agreement were calculated to give another estimate of accuracy^30^. We used their limits of agreement method to calculate 95% confidence intervals. We used this and our published pilot study 3.8 mmHg standard deviation of differences to calculate sample size of 27 subjects was required to have 80% power to detect a 95% confidence interval of ± 2.5 mmHg.
